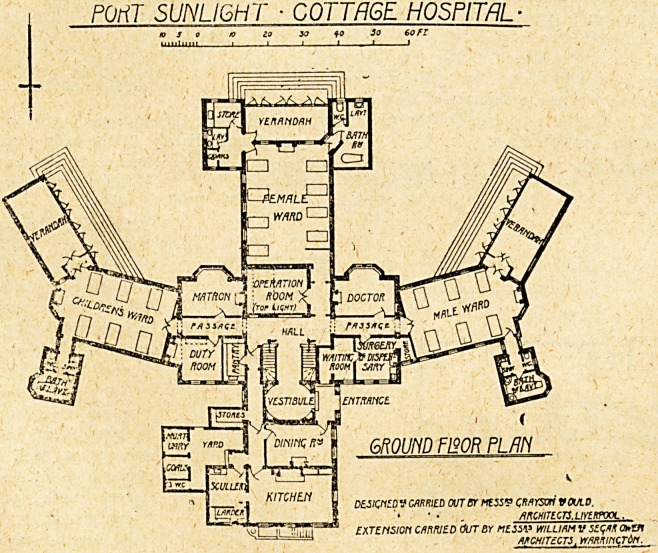# Port Sunlight Cottage Hospital

**Published:** 1916-11-04

**Authors:** 


### PORT SUNLIGHT COTTAGE HOSPITAL.

As originally planned, the cottage hospital for Messrs. Lever Brothers' factory and village of Port Sunlight comprised two ward wings, and in the centre of the south front, where is now the operation-room, a spacious glass-covered verandah was placed. It will be seen by the plan that a third ward wing now projects out from the centre, and the building takes roughly the form of a cross instead of its former T shape.

The main entrance is at the angle formed by the north and west wings. An open porch gives access on one side to a waiting-room for out-patients, on the other to a dining-room, presumably for the staff, and in the centre to a vestibule which leads into the central hall, off which the wards and other offices are arranged.

The central block, which is two storeys high, contains on the ground floor two sitting-rooms facing south, one for the resident medical officer, the other for the matron, with the operation-room and passage to the south ward block between. The operation-room has one small vertical window on the east front, but is dependent for its main lighting on a skylight the whole width of the room facing east. There is no room for the administration of anæsthetics, neither is there any separate room for sterilising or for washing-up. On the north side of this block is the ward duty-room, which serves for all three wards, a pantry, the surgery and dispensary, with waiting-room before referred to. North of the vestibule is a one-storey building containing the dining-room and kitchen offices. A small mortuary is placed in a corner of the open yard attached to the kitchen.

There are in all three wards, one of eight beds for women, one of six beds for men, and a similar one of six beds for children. Each ward has its sanitary offices and bathroom in a projecting wing with cut-off lobby. The sink-rooms are very small in the two older wings, a mistake which has been rectified in the new wing to the south.

Each ward is provided with a spacious verandah, one side of which, facing south, can be thrown entirely open, thus providing what are practically open-air wards.

In the central hall are two staircases, one of which gives access to the quarters for the resident medical officer, the other to those for the matron and some of the nurses. The remainder of the nursing staff are housed in a nursing home in the village.

The architects for the original building were Messrs. Grayson and Ould, of Liverpool, and for the extension Messrs. William and Segar Owen, of Warrington.

## Figures and Tables

**Figure f1:**